# Comparative genomics of prevaccination and modern *Bordetella pertussis *strains

**DOI:** 10.1186/1471-2164-11-627

**Published:** 2010-11-11

**Authors:** Marieke J Bart, Marjolein van Gent, Han GJ van der Heide, Jos Boekhorst, Peter Hermans, Julian Parkhill, Frits R Mooi

**Affiliations:** 1 Laboratory for Infectious Diseases and Screening, Netherlands Centre for Infectious Diseases Control, RIVM, Bilthoven, Netherlands; 2 UMC St Radboud Hospital, Nijmegen, The Netherlands; 3 Bioinformatics, Department of Biology, Faculty of Science, Utrecht University, The Netherlands; 4 The Wellcome Trust Sanger Institute, Hinxton, Cambridge, UK

## Abstract

**Background:**

Despite vaccination since the 1950s, pertussis has persisted and resurged. It remains a major cause of infant death worldwide and is the most prevalent vaccine-preventable disease in developed countries. The resurgence of pertussis has been associated with the expansion of *Bordetella pertussis *strains with a novel allele for the pertussis toxin (Ptx) promoter, *ptxP3*, which have replaced resident *ptxP1 *strains. Compared to *ptxP1 *strains, *ptxP3 *produce more Ptx resulting in increased virulence and immune suppression. To elucidate how *B. pertussis *has adapted to vaccination, we compared genome sequences of two *ptxP3 *strains with four strains isolated before and after the introduction vaccination.

**Results:**

The distribution of SNPs in regions involved in transcription and translation suggested that changes in gene regulation play an important role in adaptation. No evidence was found for acquisition of novel genes. Modern strains differed significantly from prevaccination strains, both phylogenetically and with respect to particular alleles. The *ptxP3 *strains were found to have diverged recently from modern *ptxP1 *strains. Differences between *ptxP3 *and modern *ptxP1 *strains included SNPs in a number of pathogenicity-associated genes. Further, both gene inactivation and reactivation was observed in *ptxP3 *strains relative to modern *ptxP1 *strains.

**Conclusions:**

Our work suggests that *B. pertussis *adapted by successive accumulation of SNPs and by gene (in)activation. In particular changes in gene regulation may have played a role in adaptation.

## Background

The genus Bordetella comprises nine species, of which four are exclusively respiratory pathogens of mammalian hosts: *Bordetella bronchiseptica, Bordetella parapertussis, Bordetella pertussis *and *Bordetella holmesii *[1]. The first three species are closely related, while *B. holmesii *forms a distinct branch [2]. *B. bronchiseptica *causes chronic and often asymptomatic respiratory tract infections in a wide variety of mammals and is only sporadically isolated from humans. *B. parapertussis *consists of two distinct lineages, designated *B. parapertussis_HU _*and *B. parapertussis_OV_*, which infect humans and sheep respectively [3,4]. *B. parapertussis_HU _*and *B. pertussis *are exclusive human pathogens and the causative agents of pertussis or whooping cough. Both these species have evolved independently from a *B. bronchiseptica*-like ancestor, a process which has been accompanied by extensive gene loss [4-6].

By far, most cases of whooping cough are caused by *B. pertussis*. Despite widespread vaccination, pertussis remains a major cause of infant death worldwide [7]. In the 1990s a resurgence of pertussis was observed in several countries with highly vaccinated populations and pertussis has become the most prevalent vaccine-preventable disease in developed countries [8-10]. In the Netherlands, the estimated rate of infection was 6.6% per year for the 3-79-year age group from 1995 through 1996 [11]. Similar percentages have been found in the United States [12-14]. One of the hallmarks of the pertussis resurgence is a shift in disease prevalence towards older persons who have waning vaccine-induced immunity, while recently vaccinated infants are well protected [15]. The reemergence of pertussis has been attributed to various factors including decreased vaccination coverage due to concerns over side effects, suboptimal vaccines, waning vaccine-induced immunity, and adaptation of *B. pertussis *[1,9,10]. The relative contribution of these factors may differ between countries and is the subject of ongoing debate. Pathogen adaptation is supported by several observations. We and others have shown that antigenic divergence has occurred between vaccine strains and clinical isolates with respect to surface proteins which confer protective immunity; pertussis toxin (Ptx), pertactin and fimbriae [1,16-18]. Further, in a mouse model, pertussis vaccines were less effective against strains carrying non-vaccine type antigens compared to strains with vaccine-type antigens [19-22]. Recently we found evidence that polymorphism in the promoter for Ptx (*ptxP*) may also be important in adaptation [23]. In the last twenty years two *ptxP *alleles, *ptxP1 *and *ptxP3*, predominated in the Dutch *B. pertussis *population. The *ptxP3 *strains were first observed in 1988, gradually increased in frequency, and nearly completely replaced the resident *ptxP1 *strains in the late 1990s. In the Netherlands, the increase in frequency of *ptxP3 *strains was associated with the resurgence of pertussis. The *ptxP3 *strains are found in Asia, Europe, North and South America, and there is evidence that they have spread worldwide in the 1980s and 1990s [23]. The *ptxP3 *strains produced more Ptx than the *ptxP1 *strain and epidemiological data suggest that *ptxP3 *strains are more virulent. Ptx suppresses both the innate and adaptive immune system [24,25] and we have proposed that increased Ptx production increases pathogen fitness in vaccinated populations by enhancing transmission by hosts in which vaccine immunity has waned. Thus, both antigenic divergence and increased immune suppression in combination with waning immunity are likely to contribute to the pertussis resurgence [23]. Here we extend our studies on adaptation of *B. pertussis *using comparative genomics. We determined, annotated and compared genome sequences of six Dutch strains, two of which were isolated before vaccination was introduced in 1953 and four modern strains, isolated approximately 50 years later. The modern strains carried either the *ptxP1 *allele or the *ptxP3 *allele, while the pre-vaccination strains carried *ptxP1 *or *ptxP2*. We identified novel polymorphisms in specific genes and gene categories which may play a role in the persistence and resurgence of pertussis in the face of intensive vaccination.

## Results and Discussion

### Strain selection

Our long term aim is to identify *B. pertussis *loci which have contributed to the persistence and resurgence of pertussis in vaccinated populations. We selected two strains isolated in 1949 and 1952 which were characteristic for the Dutch *B. pertussis *population in the pre-vaccine era [16,26], and four modern strains isolated in 1999 and 2000, approximately 50 years after the introduction of vaccination. Two of the modern strains carried the *ptxP1 *allele and two the *ptxP3 *allele. In our comparisons, we included the Tohama I strain (*ptxP1*) of which the annotated genome sequence was available [6]. The Tohama I strain was isolated in Japan in the 1950s and has been subcultured in vitro extensively. It is used as model strain in many different laboratories. Strain characteristics are listed in Table 1.

**Table 1:** Isolates used in this study^1^

Isolate	Coverage	Genome size (Mb)	Country	Year	Sero type	*ptxP*	*ptxA*	*prn*	*fim2*	*fim3*	Ref
Tohama I	NA	4,09	Japan	1952	2	*ptxP1*	*ptxA2*	*prn1*	*fim2-1*	*fim3-1*	[6]

B0558	16×	4,12	Netherlands	1949	3	*ptxP1*	*ptxA2*	*prn1*	*fim2-1*	*fim3-1*	this work

B1193	15×	4,12	Netherlands	1950	2,3	*ptxP2*	*ptxA4*	*prn7*	*fim2-2*	*fim3-1*	this work

B1831	28×	4,08	Netherlands	1999	3	*ptxP3*	*ptxA1*	*prn2*	*fim2-1*	*fim3-2*	this work

B1834	28×	4,08	Netherlands	1999	3	*ptxP1*	*ptxA1*	*prn2*	*fim2-1*	*fim3-1*	this work

B1917	15×	4,08	Netherlands	2000	3	*ptxP3*	*ptxA1*	*prn2*	*fim2-1*	*fim3-2*	this work

B1920	15×	4,08	Netherlands	2000	3	*ptxP1*	*ptxA1*	*prn2*	*fim2-1*	*fim3-1*	this work

^1 ^Year of isolation and alleles of main virulence factors are shown. Coverage refers to oversampling in sequence data. Genome size was based on size of the Tohama I genome corrected for the large regions of differences. Abbreviations: Mb, megabases; NA not available.

### Comparative analysis of *Bordetella pertussis *genomes

We determined the genome sequences of the six *B. pertussis *strains through pyrosequencing. As this technology is known to generate errors in homopolymeric nucleotide tracts, SNPs and indels in these regions were filtered out. As a consequence, differences between strains in homopolymeric nucleotide tracts were not identified. However, homopolymeric nucleotide tracts have high mutation rates and may vary during subculturing of a single strain [27]. Thus, genotypes and phenotypes controlled by homopolymeric nucleotide tracts are not stable and changes in these tracts will not represent fixed differences between strains.

We identified 471 SNPs (i.e. bases that were not conserved in one or more of the seven strains), of which 414 and 57 were located in ORFs and intergenic regions, respectively (Additional file 1). Four ORFs were found to contain small insertion or deletions (indels) ranging from eight to 31 bases (Additional file 1). Based on our analyses, the estimated SNP density was 1 SNP per 8,675 bases. Maharjan and coworkers used Microarray-based comparative genome sequencing to detect SNPs in 34% of the Tohama I genome [28]. The Tohama I strain was compared to an Australian isolate from 2006 and a SNP density of 1 SNP per 20,000 bases was found. As we included the whole genome in our comparison and used a larger number of strains, the higher SNP density we found was not unexpected. SNP densities in other monomorphic human pathogens have been found to range from 1 SNP per 2,300 bases in *Salmonella enterica serovar Typhi *[29] to 1 SNP per 28,400 bases in *Mycobacterium leprae *[30,28]. This places *B. pertussis *among the most monomorphic human pathogens known. In their analyses, Maharjan and coworkers identified 66 SNPs in 1,229 genes and 4 SNPs in 268 intergenic regions. Of these 70 SNPs, 27 (39%) were also detected in one of the six Dutch strains, while 14 (20%) were specific for the Australian strain.

In addition to the SNPs discussed above, we confirmed 13 large regions of difference (RDs) identified in previous studies using microarrays [31-35] and whole genome sequencing [33,36]. Further, we found a new polymorphism in RD23 (Additional files 2 and 3).

### Genetic relationship based on whole genome sequencing

**Figure 1:**
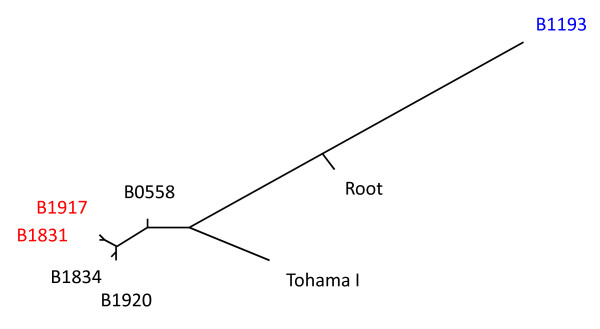
**Phylogenetic tree of strains used in this study based on 471 SNPs**. Colored strain designations indicate *ptxP *alleles: black, *ptxP1*; blue, *ptxP2; *red, *ptxP3*. The location of the root was based on the sequence of *B. bronchiseptica *RB50 strain [6].

A maximum likelihood phylogenetic tree based on 471 SNPs was derived (Figure 1). The genome sequence of *B. bronchiseptica *was used as outgroup to root the tree [6]. The tree suggested that B1193, which harbors the *ptxP2 *allele, represented a distinct lineage which diverged from the six other strains relatively early in the history of *B. pertussis*. Although the Tohama I strain was more closely related to the four remaining Dutch strains, it also formed a distinct, relatively deep branch. This may be due to the fact that the Tohama I strain has been subcultured in vitro since the 1950s resulting in a relaxation of selective forces, or it could reflect geographical isolation, as the Tohama I strains was isolated in Japan, while all other strains were from the Netherlands. The four recently isolated Dutch strains were closely related. The tree indicated that the *ptxP3 *strains comprise a monophyletic lineage which recently diverged from modern *ptxP1 *strains.

### Evidence for selection in cis regulatory regions

**Figure 2:**
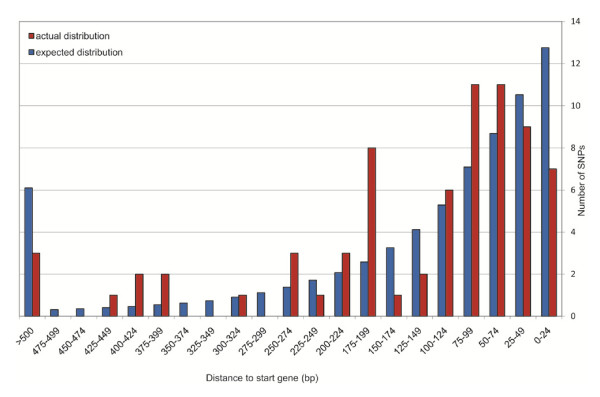
**Distribution of SNPs in intergenic regions relative to the start codons of predicted protein coding genes**. The number of SNPs was plotted as a function of the distance to the initiation codon of genes using a window of 25 bases. Blue columns show the expected distribution based on the frequency distribution of 25 bases segments in intergenic regions. Red columns show the actual distribution. The two distributions are significantly different (Chi-square test, P 0.01).

Although it is often assumed that mutations located outside protein coding sequences (CDSs) are neutral, such mutations can affect fitness if they are located in cis-regulatory elements (i.e. in regions involved in binding of transcriptional factors or in regions that affect translation). To explore the role of cis regulatory elements in adaptation, we determined the distribution of all SNPs in intergenic regions relative to the start codon of predicted CDSs in segments of 25 bases (Figure 2). Assuming random mutation (the null hypothesis), one expects a SNP distribution which reflects the frequency of the 25 base intergenic segments in the chromosome. This frequency is highest for the proximate 25 base segment and decreases with distance to the start codon (Figure 2). The actual frequency distribution was compared with the frequency based on null hypothesis and found to be significantly different (P 0.01). In view of the low number of SNPs observed, the region 200 to > 500 will be ignored. In the regions 50-99 and 175-199 more SNPs were observed than in the random mutation model, suggesting diversifying selection. These regions may contain binding sites for regulatory proteins such as BvgA, as shown for Ptx [37]. The regions 125-174 and 0-24 contained less SNPs compared to the random model, possibly reflecting purifying selection. The region 0-24 contains the binding site for RNA-polymerase and regions involved in initiation of translation and it seems that such regions tend to be conserved in *B. pertussis*. Thus our results provide evidence that regions involved in binding of regulatory proteins are subject to diversifying selection suggesting a role in adaptation. The low degree of polymorphism, the limited number of strains sequenced and the absence of silent mutations in some CDSs did not allow calculation of dN/dS ratios to identify purifying or diversifying selection in ORFs [38-40].

### Polymorphisms in pathogenicity-associated genes

**Figure 3:**
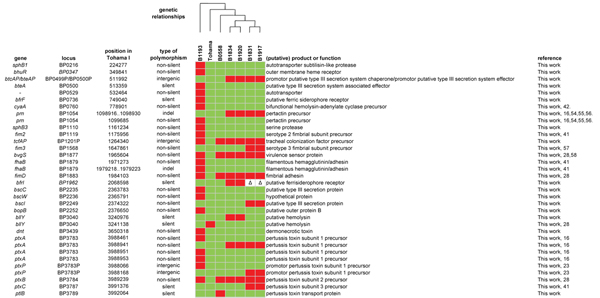
**Polymorphisms found in virulence-associated genes**. The distribution of polymorphisms over the analyzed strains is shown. Within rows, polymorphisms are distinguished by color. BP1962 is part of RD10 which is deleted from *ptxP3 *strains (See Fig. 6). Deleted genes are indicated by "Δ". An overview of all known alleles is given in Additional file 5. Abbreviation: indel, insertion/deletion. References [16,23,28,41,42,54-58].

The 89 pathogenicity-associated genes (Additional file 4) contained 34 polymorphisms (SNPs and small indels) distributed over 24 genes and three promoter regions (Figure 3 and Additional file 5). To the best of our knowledge, 16 of the polymorphisms in pathogenicity-associated genes were not described before. Nineteen of the 34 polymorphisms were specific for the *ptxP2 *strain, again underlining the distinct nature of this strain. It was noteworthy that for Ptx, Prn (unpublished data), the type III secretion toxin (BteA) and tracheal colonization factor (TcfA), polymorphisms were found in both the protein coding sequences [41,42] and the (putative) promoter regions, suggesting both structural and regulatory adaptations for these virulence factors.

### Polymorphisms which distinguish strains isolated before and after the introduction of vaccination

**Figure 4:**
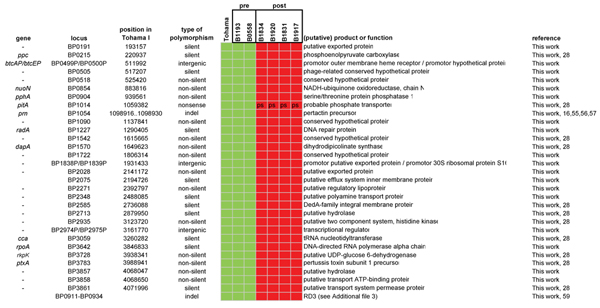
**Polymorphisms which distinguish strains isolated before (pre) and after (post) the introduction of vaccination**. Within rows, polymorphisms are distinguished by color. Genes truncated in all strains were omitted. An overview of alleles is given in Additional file 6. Abbreviation: indel, insertion/deletion. Ps, pseudogene. References [16,28,55-57,59].

**Figure 5:**
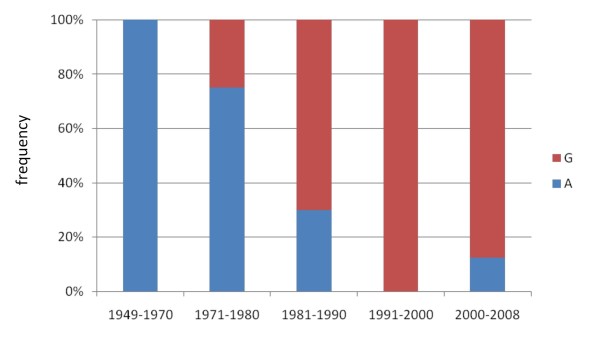
**Temporal trend in the frequency of a SNP located in the promoter region for the type III secretion system**. The SNP involved an A/G transition and was located between the type III secretion toxin gene *bteA *and its chaperone *btcA*. The two genes are oriented back to back. The period 1949-2008 was investigated using 44 strains.

The comparison of polymorphisms which distinguish strains isolated before and after the introduction of vaccination (for convenience designated as pre- and post-strains) may be useful to identify mutations which increase fitness in vaccinated populations and hence reduce vaccine efficacy. We identified 14 non-silent, 11 silent SNPs, three intergenic SNPs, one nonsense mutation and two indels which distinguished pre- and post-strains (pseudo-genes were excluded). The distribution of alleles and SNPs are shown in Additional file 6 and Figure 4, respectively. Here, only polymorphisms believed to be particularly interesting based on our current knowledge are discussed. The post-strains were distinguished by a large deletion (RD3) comprising 24 CDSs. King et al. [34] analyzed a larger number of post-strains (N = 43), all of which were found to miss RD3. RD3 contains four ORFs coding for putative exported proteins and its removal may reduce the antigenic profile of *B. pertussis*. Alternatively, the deletion of this region may be unrelated to vaccination and reflect the ongoing genome reduction [32,35]. One gene, *pitA *coding for a putative phosphate transporter, was inactivated in post-strains by a nonsense mutation. The nonsense mutation was also observed in an Australian strain [28]. Post-strain-specific, non-silent, polymorphisms were found in two genes for exported virulence factors, *prn *and *ptxA*, in agreement with previous studies [18]. There is evidence that polymorphism in these two genes affects vaccine efficacy in a mouse model [19-22]. The locus for another (putative) exported protein (BP2028) also contained a non-silent polymorphism specific for post-strains. Blast searches revealed 100% identity at the amino acid level between the prevaccination variant and homologues found in *B. bronchiseptica *and *B. parapertussis*, suggesting that the prevaccination strains harbor the ancestral allele. No significant similarity was found with CDSs from other bacteria, suggesting a highly specific function. Although the function of the protein is not known, the fact that it is exported suggests a role in host-pathogen interaction. Also of interest is a SNP in the promoter region for the type III secretion toxin and chaperone genes, *bteA *and *btcA *respectively. For *B. bronchiseptica *it has been shown that BteA is translocated into the host and is necessary and sufficient for rapid cytotoxicity in a wide range of mammalian cells [43,44]. The SNP, which involves an A/G transition, is located in a putative TATA box of *btcA *and therefore could influence its expression. We investigated the temporal trends of the two alleles, designated allele A and allele G (Figure 5). Allele A was found in all strains from 1949 to 1970 (N = 12). Allele G was first observed in 1977 and gradually increased in frequency to ~90% in 2000-2008. The allele A was found in *B. bronchiseptica *suggesting that it is the ancestral type, consistent with the trend observed in the Netherlands.

### Polymorphisms which distinguish *ptxP3 *strains

**Figure 6:**
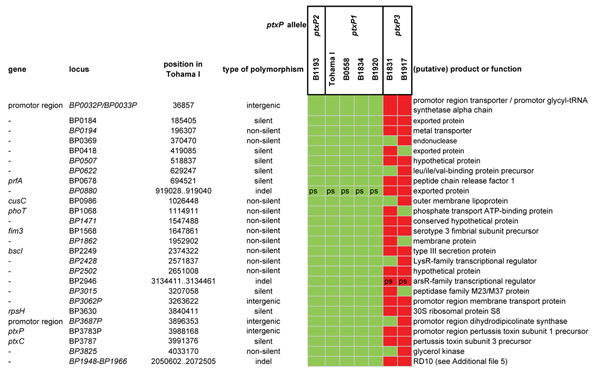
**Polymorphisms associated with *ptxP3 *strains**. The distribution of polymorphisms over the analyzed strains is shown. Within rows, polymorphisms are distinguished by color. Genes truncated in all strains were omitted. An overview of all known alleles is given in Additional file 7. Abbreviation: indel, insertion/deletion; ps, pseudogene.

Strains carrying the *ptxP3 *allele have spread worldwide and have contributed to the pertussis epidemic in The Netherlands [23]. It seems likely that the *ptxP3 *allele confers increased fitness compared to the *ptxP1 *allele it replaced in current human populations. Alternatively, it may be a marker for other selective changes in the same haplotype. To identify other genes that may have contributed to the increased fitness of the *ptxP3 *lineage, we sought polymorphisms which were unique for the *ptxP3 *lineage. We identified 26 polymorphisms specific for one or both *ptxP3 *strains, 11 non-silent SNPs, eight silent SNPs, four intergenic SNPs, two small indels (eight and 31 bases) and one large deletion (RD10, comprising 2.2 kbases). The distribution of alleles and polymorphisms are shown in Additional file 7 and Figure 6, respectively. Only polymorphisms we believe to be particularly interesting based on our current knowledge are discussed. The association of the RD10 deletion with *ptxP3 *strains was noted before using a larger collection of strains (N = 15) [34]. One of the intergenic SNPs was located in the *ptx *promoter and defines the *ptxP3 *lineage. The remaining three intergenic SNPs were located proximal to the genes for glycyl tRNA synthetase alpha chain gene (*glyQ*), a transporter, an integral membrane transport protein and a dihydrodipicolinate synthetase. It is not clear whether these SNPs affect transcription. We have shown previously that the SNP in the Ptx promoter region affects production of Ptx, however [23]. Two *ptxP3*-specific SNPs were in known virulence genes, *fim3 *and *bscI*, coding for the serotype 3 fimbriae and a component of the type III secretion system, respectively [43,45]. Two indels, located in BP0880 and BP2946, were *ptxP3-*specific. BP0880 codes for a putative exported protein which shows similarity with metal dependent phosphohydrolases and signal transduction proteins, while the BP2496 product belongs to the family of *arsS *transcriptional regulators. BP0880 was a pseudogene in all strains, except the two *ptxP3 *strains in which the reading frame was restored by deletion of an eight base repeat. Conversely, the reading frame of *arsS *was intact in all strains except the two *ptxP3 *strains, due to the deletion of a 31 base repeat. Both BP0880 and BP2496 are intact in *B. bronchiseptica *and *B. parapertussis*. The BP0880 and BP2496 genes from three *ptxP3 *strains were sequenced completely and the ORFs were found to be intact and truncated, respectively. It seems likely that BP0880 and BP2946 are subject to phase variation, i.e. the reversible on/off switching of genes by varying DNA sequence repeat units [46]. Phase variation has been observed for a number of other *B. pertussis *genes [27,45,47]. Based on the phylogenetic relationships, it seems likely that BP0880 and BP2949 were, respectively, reactivated and inactivated in the *ptxP3 *lineage. This qualifies these loci for further analyses as to their role in the emergence of *ptxP3 *strains.

## Conclusions

We provide the first comprehensive genomic comparison of a bacterial pathogen circulating in a highly vaccinated population. In this study, we included two strains from the prevaccination era and four strains isolated ~50 years later. We confirmed and extended the observation that modern *B. pertussis *strains differed significantly from prevaccination strains, both phylogenetically and with respect to particular alleles [1]. Further, we identified one highly divergent, possibly ancient, *B. pertussis *lineage, characterized by the *ptxP2 *allele. Our work confirmed that *B. pertussis *strains differ significantly in gene content due to gene loss, a process which may still be ongoing [32,35]. Further, we found no evidence that acquisition of novel genes has played a role in adaptation, as has been suggested for *B. holmesii *[2]. In contrast, *B. pertussis *seems to adapt mainly by the successive accumulation of SNPs. Our work shows that, based on SNP density, *B. pertussis *is one of the most monomorphic human pathogens. This suggests a recent origin of this species or, more likely, a recent population bottle neck [1,4].

Our results provide evidence that regions involved in binding of regulatory proteins are subject to diversifying selection suggesting a role in adaptation. Indeed, this is exemplified by the rapid emergence of *ptxP3 *strains with increased pertussis toxin production [23]. A number of recent studies have highlighted the importance of changes in gene regulation in adaptation of pathogens [48-51]. It is noteworthy that for a number of virulence factors (Ptx, Prn and TcfA), SNPs were found in both the protein-encoding genes and the (putative) promoter regions, suggesting adaptation at both the structural and regulatory level for the same phenotype. Of interest is a SNP in the promoter region for the type III secretion toxin (BteA) and chaperone genes (BtcA). For *B. bronchiseptica *it has been shown that BteA is necessary and sufficient for rapid cytotoxicity in a wide range of mammalian cells [43,44]. The ancestral allele was found in all strains from before 1977, but subsequently replaced by a novel allele which increased in frequency to ~90% in 2000-2008. This suggests that the novel allele may significantly affect strain fitness, although it is also possible that its increase in frequency is due to hitchhiking with other loci which affect fitness. In any case, this allele may be an important marker for successful lineages. The *ptxP3 *lineage, associated with the resurgence of pertussis in the Netherlands, has emerged recently and spread worldwide [23]. We found that the *ptxP3 *strains comprised a young branch which diverged recently from modern *ptxP1 *strains. Several alleles were identified, which were uniquely associated with the *ptxP3 *lineage and may thus have contributed to its success. Two *ptxP3*-specific SNPs were in known virulence genes, *fim3 *and *bscI*, coding for the serotype 3 fimbriae and a component of the type III toxin secretion system, respectively. We also observed both reactivation and inactivation of genes in the *ptxP3 *lineage. In conclusion, this work has identified a number of genetic loci which are associated with highly successful strains. Further analyses of these loci can contribute to our understanding of the evolution of bacterial pathogens.

## Methods

### Strain, culture conditions and DNA isolation

The six clinical isolates used in this study are described in Table 1. Strains were grown on Bordet Gengou (BG) agar supplemented with 15% sheep blood and incubated for 3 days at 35°C. DNA was isolated using QIAGEN Genomic-tip 100/G kit, according to the manufacturer's instructions.

### Genome sequencing and detection of polymorphisms

B1831 was sequenced using the 454 GS-G20 sequencer (Roche) and the other five isolates were sequenced using the 454 GS-FLX sequencer (Roche), according to the manufacturer's instructions. The generated reads were assembled *de novo *into contigs using the Newbler assembler (Roche).

SNPs, insertions and deletions were detected by mapping the contigs to the previously sequenced and annotated *B. pertussis *Tohama I genome [6] using BLAST. We filtered SNP calls as described in [29]. Briefly, SNPs with low base quality, SNPs within 15 base of the end of a contig, SNPs in repetitive sequences, such as insertion sequence elements, and SNPs in homopolymeric tracts were removed. Single base insertions or deletions in homopolymeric tracts were ignored as these are often a result of 454 sequencing errors [52]. To determine the accuracy of these SNPs we verified 88 SNPs in the sequenced strains by combining PCR with mass spectrometry (Sequenom). All 88 SNPs were correct. Only for a subset of small indels (≥ 6 b), which distinguished sets of strains, were checked by resequencing and included in the analyses. Genome sequences have been submitted to the NCBI Nucleotide Sequence data base (accessions numbers; strain B0558, ADKR00000000; strain B1193, ADKS00000000; strain B1831, ADKT00000000; strain B1834, ADKU00000000; strain B1917, ADKV00000000; strain B1920, ADKW00000000).

### Phylogenetic analysis

Only SNPs for which the allele at the orthologous nucleotide was determined in all strains were included in the phylogenetic analysis. A Maximum Likelihood tree was derived using PhyML [53] with the following parameters: model, HKY; transition/transversion ratio; estimated; proportion of invariable sites, estimated; number of relative substitution rate categories, 4; gamma distribution parameter, estimated; starting tree, BIONJ; optimize tree topology, yes; optimize branch lengths and rate parameters, yes.

### Statistical analyses

To determine if there is evidence for selection in cis-regulatory loci, we compared the expected distribution of the distance of SNPs to the start of the gene with the actual distribution of these distances using the Chi square test. This was done for all genes.

## Authors' contributions

MB carried out the molecular genetic studies, analyzed the data and contributed to discussions and writing of the manuscript. MvG carried out the molecular genetic studies, analyzed the data and contributed to discussions and writing of the manuscript. HvdH analyzed the data. JB analyzed the data and contributed to discussions and writing of the manuscript. PH contributed to discussions and writing of the manuscript. JP contributed to discussions and writing of the manuscript. FM conceived the project and wrote the first draft of the manuscript. All authors read and approved the final manuscript.

## Supplementary Material

Additional file 1 **Supplementary table S1A & S1B & S1C.xlsx**. Contains a list of SNPs, small insertions and deletions found in the analyzed genomes. Click here for file

Additional file 2 **Supplementary figure S1.jpg**. Shows large regions of differences (RDs) between six Dutch *B. pertussis *strains and Tohama I Click here for file

Additional file 3 **Supplementary table S2.xlsx**. Shows large Regions of Difference (RDs) between the analyzed genomes. Click here for file

Additional file 4 **Supplementary table S3.xlsx**. Shows functional gene categories. Click here for file

Additional file 5 **Supplementary table S4.xlsx**. Shows allelic polymorphisms found in pathogenicity-associated genes Click here for file

Additional file 6 **Supplementary table S5.xlsx**. Shows alleles which distinguish strains isolated before and after the introduction of vaccination Click here for file

Additional file 7 **Supplementary table S6.xlsx**. Shows alleles unique for one or both *ptxP3 *strains Click here for file
