# Supplementary material for: Comparative genomics of prevaccination and modern Bordetella pertussis strains
Source: BMC Genomics. 2010 Nov 11;11:627. doi: 10.1186/1471-2164-11-627 (PMC3018138; doi:10.1186/1471-2164-11-627)
Supplement: Additional file 2 — Supplementary figure S1.jpg. Shows large regions of differences (RDs) between six Dutch B. pertussis strains and Tohama I [file 1471-2164-11-627-S2.PPTX]

## Slide 1
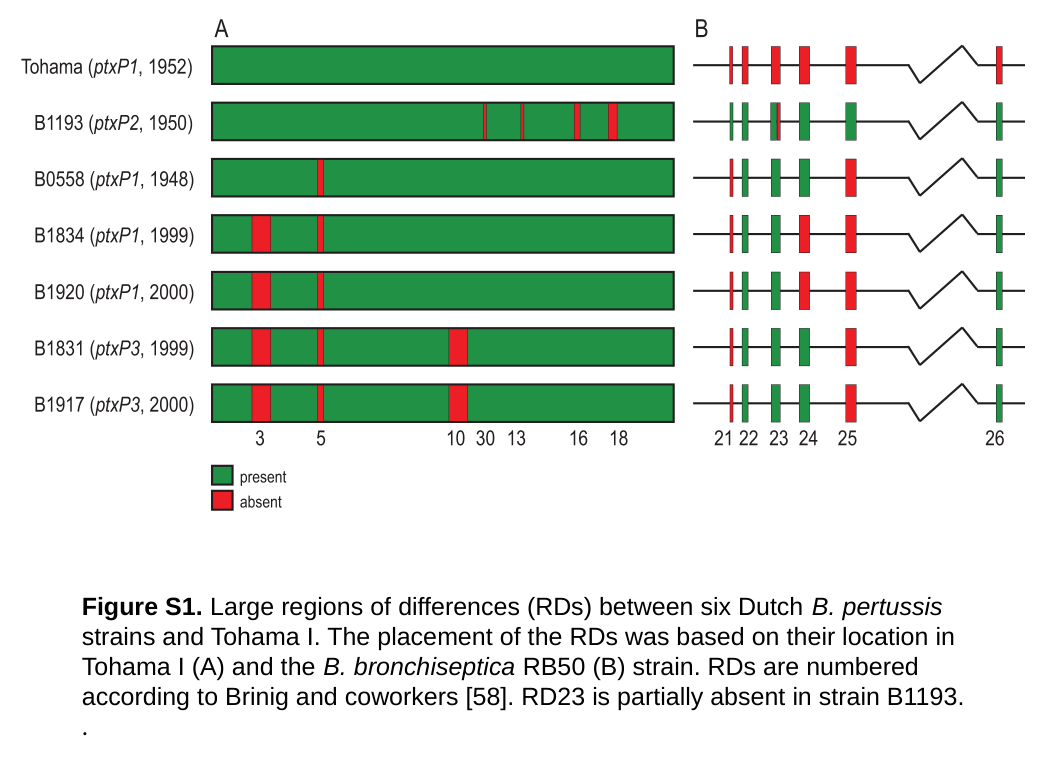

Figure S1. Large regions of differences (RDs) between six Dutch B. pertussis strains and Tohama I. The placement of the RDs was based on their location in Tohama I (A) and the B. bronchiseptica RB50 (B) strain. RDs are numbered according to Brinig and coworkers [58]. RD23 is partially absent in strain B1193.
.
